# Sleep EEG-Based Approach to Detect Mild Cognitive Impairment

**DOI:** 10.3389/fnagi.2022.865558

**Published:** 2022-04-13

**Authors:** Duyan Geng, Chao Wang, Zhigang Fu, Yi Zhang, Kai Yang, Hongxia An

**Affiliations:** ^1^State Key Laboratory of Reliability and Intelligence of Electrical Equipment, Hebei University of Technology, Tianjin, China; ^2^Key Laboratory of Electromagnetic Field and Electrical Apparatus Reliability of Hebei Province, Hebei University of Technology, Tianjin, China; ^3^Physical Examination Center, The 983 Hospital of Joint Logistics Support Force of the Chinese People’s Liberation Army, Tianjin, China

**Keywords:** mild cognitive impairment, sleep EEG, sleep slow waves, sleep spindles, machine learning

## Abstract

Mild Cognitive Impairment (MCI) is an early stage of dementia, which may lead to Alzheimer’s disease (AD) in older adults. Therefore, early detection of MCI and implementation of treatment and intervention can effectively slow down or even inhibit the progression of the disease, thus minimizing the risk of AD. Currently, we know that published work relies on an analysis of awake EEG recordings. However, recent studies have suggested that changes in the structure of sleep may lead to cognitive decline. In this work, we propose a sleep EEG-based method for MCI detection, extracting specific features of sleep to characterize neuroregulatory deficit emergent with MCI. This study analyzed the EEGs of 40 subjects (20 MCI, 20 HC) with the developed algorithm. We extracted sleep slow waves and spindles features, combined with spectral and complexity features from sleep EEG, and used the SVM classifier and GRU network to identify MCI. In addition, the classification results of different feature sets (including with sleep features from sleep EEG and without sleep features from awake EEG) and different classification methods were evaluated. Finally, the MCI classification accuracy of the GRU network based on features extracted from sleep EEG was the highest, reaching 93.46%. Experimental results show that compared with the awake EEG, sleep EEG can provide more useful information to distinguish between MCI and HC. This method can not only improve the classification performance but also facilitate the early intervention of AD.

## Introduction

As the aging of the population becomes increasingly serious, Alzheimer’s disease has become a major challenge to human health and a serious social problem. Alzheimer’s disease (AD) is the most common type of dementia, accounting for roughly 70% of all dementias worldwide. It is an irreversible neurodegenerative disease marked by cognitive, behavioral, and intellectual impairments (Prince, 2015). Mild cognitive impairment (MCI) is a pre-dementia condition in which daily functioning is usually maintained despite objectively measured cognitive impairment in one or more cognitive domains. As MCI is the primary stage of cognitive impairment, about 10–15% of MCI patients will progress to AD every year on average, and about 2/3 of AD patients are developed from MCI (Tsai et al., 2016). As a result, early detection of MCI is critical for early intervention in the preclinical stage of AD and has attracted much attention from researchers in recent decades (Jiang et al., 2020).

According to recent studies, patients with MCI may return to normal over time, therefore early detection and diagnosis of MCI are critical (Amezquita-Sanchez et al., 2019). Early detection of cognitive decline can lead to appropriate interventions before further cognitive impairment occurs, thus delaying or even preventing the progression of dementia as much as possible. It is estimated that the average annual cost per patient for mild dementia is $15,889, for moderate dementia is $26,859, and for severe dementia is $36,180. Prevention of the disease is therefore important for better health care, as well as for national financial interests, and for controlling the progression of cognitive impairment.

Finding biomarkers with low cost, high specificity, and sensitivity has been the focus of MCI research. Magnetic resonance imaging, such as functional magnetic resonance imaging (fMRI) (Ni et al., 2017), magnetic resonance spectral imaging (MRS) (Gao and Barker, 2014), diffusion-weighted imaging (DWI) (Ge, 2017), diffusion tensor imaging (DTI) (Ahmed et al., 2017), positron emission tomography, such as fluorodeoxyglucose positron emission tomography (FDG-PET) (Karow et al., 2010), etc., cerebrospinal fluid markers (Handels et al., 2017), such as Aβ40, Aβ42, total tau protein (t-tau), etc., are currently the main methods for early diagnosis of MCI. However, these methods are expensive, and the equipment is large, with high expertise requirements. As a result, researchers are looking for non-invasive, quick, low-cost, and dependable approaches for disease detection (Alberdi et al., 2016).

Biomarkers based on EEG have emerged as a viable tool in the research of AD. In terms of EEG acquisition methods, most of the published work relies on the analysis of closed resting state EEG (rsEEG) recordings. Waninger et al. (2016) used fast Fourier transform to calculate Power Spectral Density (PSD) to study the difference between MCI and HC and found that there were significant differences in theta and alpha frequency bands, which were classified by linear discriminant analysis. The final classification result was 85.11%. Rodrigues et al. (2021) conducted statistical analysis of lacstral distances between EEG subbands and found a metric that could identify AD at all stages and characterize AD activity in each electrode, achieving a classification accuracy of 98.06% with an artificial neural network. Cassani and Falk (2020) proposed a new feature characterized by a two-dimensional modulation spectrum domain based on rsEEG signals, collected EEG signals of 20 channels, and obtained classification accuracy of 88.1% by SVM classification of MCI and HC. Other papers extract evoked potentials by giving specific stimuli to the nervous system to detect and classify MCI and other disorders that affect cognitive states. For instance, Khatun et al. (2019) proposed an MCI detection method based on single-channel EEG, which stimulated auditory speech signals, extracted features from event-related potential (ERP), and obtained an accuracy of 87.9% by SVM classification. Although the literature has reported levels of accuracy above 80%, it has been difficult to evaluate studies and determine the most advanced approaches due to variances in experimental settings, data collection methods, and database sizes.

Changes in sleep electrophysiology may be linked to the cognitive condition of AD and MCI patients, according to recent research (Gorgoni et al., 2020). Local sleep EEG oscillations have a critical function in learning and plasticity mechanisms, it’s worth highlighting. Several electrophysiological aspects of NREM (such as slow waves, sleep spindles, and hippocampal ripples) and REM sleep (such as θ activity) are particularly active in memory consolidation (Klinzing et al., 2019). Sleep EEG can identify the sleep changes associated with AD and MCI pathology, and is low-cost and portable, so it can be utilized to make quick and precise diagnoses (D’Atri et al., 2021). This is the main motivation of this study. Various studies have employed multi-channel EEG data to characterize MCI or AD using EEG signals. Although there are several EEG-based studies in the literature, no one has attempted to detect and classify MCI using two-channel sleep EEG signals, as far as we are aware. Compared with the multi-channel, the number of two-channel leads is less, and the measurement method is simple. Moreover, compared with the EEG signal during waking, the EEG signal collected during sleep is stable and easy to be disturbed, which is more conducive to the study of neurodegenerative diseases.

This study proposes a new method to distinguish the EEG signals of MCI and health control (HC). We use from the C3 and C4 (central electrode) dual channel sleep EEG data with labels, based on the sleep slow waves, spindles, power spectral density and complexity, the use of machine learning and deep learning methods classifying MCI, and the classification results were compared with the awake EEG classification results without sleep features. The paper is organized as follows: section “Materials” describes the data set used for this work, and the section “Methodology” introduces the signal processing methods, including the detection of sleep slow waves and spindles, as well as the method of feature extraction and classification. Section “Result and Discussion” is the experimental results and the discussion of the paper. Finally, the conclusions are contained in section “Conclusion.”

## Materials

### Data and Materials

All data were obtained from NSRR (Zhang et al., 2018). To balance the data, 40 subjects (20 patients with MCI and 20 healthy subjects as controls, all women) with polysomnography (PSG) signals were randomly selected in the SOF study (Spira et al., 2008). All data were approved by the local institutional review board of the institution, and each participant provided written, informed consent before participation. The data included functional tests, cognitive exams, use of medication, health habits, and much more. All subjects underwent the Mini-mental State Examination (MMSE) to determine the severity of their impairment or dementia. According to the classification method of dementia severity, the MMSE score between 21 and 26 was considered MCI, and the MMSE score greater than 26 was normal. The comprehensive demographic information of the subjects in this study is shown in Table 1.

**TABLE 1:** Corresponding statistical information of subjects.

	Subjects	Age (year)	MMSE
HC	20 (female)	82.95 ± 2.71	29.6 ± 0.73
MCI	20 (female)	84.05 ± 3.51	24.4 ± 0.97

MCI exclusion criteria are as follows:

(i) a history of depression (mild to moderate or major depression) or a history of adolescent paroxysmal mental illness; (ii) a history of major stroke or neurological symptoms; (iii) Other mental disorders, frontotemporal dementia, Lewy body dementia, vascular dementia, epilepsy, alcohol dependence; (iv) The use of psychoactive drugs, which modulate EEG markers; And (v) current or previously uncontrolled or complex systemic diseases (including diabetes), or traumatic brain injury (Moretti et al., 2013).

SOF data includes EDF and annotation files that include manually graded sleep stages in 30-s epochs, as well as manual annotations for arousal, limb movement, and signal artifacts. All experiments employed the American Academy of Sleep Medicine (AASM) staging, with NREM3 and NREM4 compressed to the N3 stage, and electrode labels were taken from the International 10-20 system.

### EEG Data and Preprocessing

EEG channels are selected from the multi-channel PSG signals, and the signals are down-sampled to 100 Hz to speed up the calculation time. A text file of the sleep stage vector (also known as hypnogram) is then loaded at a sampling frequency of 1/30 and sampled upward to match the sampling frequency and length of the EEG signal.

Sleep changes are a core component of MCI and AD patients. The decrease in slow-wave activity (SWA) during NREM is due in part to amyloid disease and leads to cognitive decline in older adults (Rosinvil et al., 2020). Furthermore, studies have revealed that patients with MCI often have a substantial decrease in spindles, which is associated with cognitive decline in dementia patients (Gorgoni et al., 2016). Because sleep quality degradation is one of the primary symptoms of MCI, a variation in EEG activity during NREM sleep could be a possible biomarker (Romanella et al., 2021). Spindles are particularly noticeable during N2 sleep and are a distinguishing feature of this stage, while sleep slow waves are present during both N2 and N3. Therefore, for the main analysis, a hypnogram was used to extract the EEG signals of the N2 and N3 stages and the EEG signals of the waking stage for comparison, so as to verify the importance of sleep features.

First, the EEG signals were bandpass filtered between 0.1 and 30 Hz, and the artifact signals were eliminated using independent component analysis (ICA). The signals of each channel are divided into 5-min non-overlapping segments, and the abnormal segment is removed using the standard deviation-based rejection method. This method is speedier and is based solely on the standard deviation distribution of each segment. First, the standard deviation of each segment and channel is determined, and the resulting array of standard deviations is log-transformed and z-scored. Any epochs that have one or more channels that surpass the threshold will be labeled as an artifact. Because this method is more sensitive to the effects of noise, any segment with overlapping wake, motion, or signal artifact annotations have been removed before using this method. Accordingly, the segment of each subject was evaluated which accounts for a total of 2063 segments for NREM sleep and a total of 768 segments for wakefulness are analyzed.

## Methodology

**FIGURE 1:**
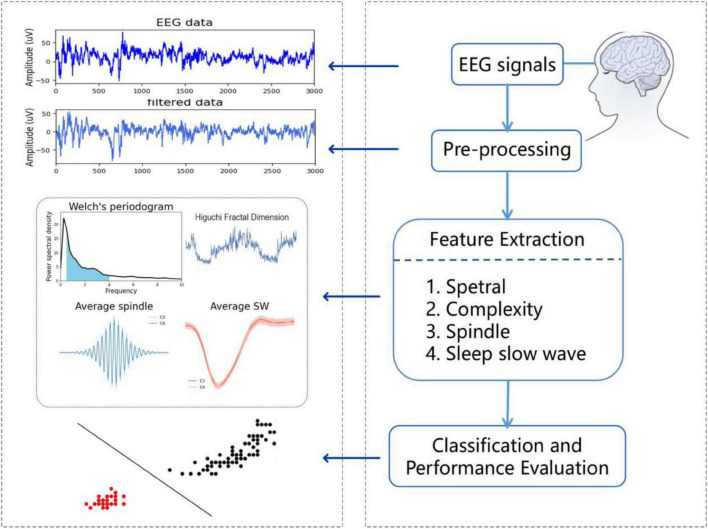
Schematic diagram of proposed algorithm.

Figure 1 describes a proposed algorithm for classifying EEG segments from MCI and HC. As shown in the figure, the algorithm consists of three steps. The first step is to calculate the power spectral density of each frequency band. For the sleep EEG signals, we mainly focus on sleep slow waves and spindles during NREM sleep, and use the YASA algorithm to detect sleep slow waves and spindles. Secondly, extract the features of sleep slow waves and spindles and calculate the spectral and complexity features. Thirdly, train the classifier with the extracted features and evaluate the test results. In addition, to confirm the validity of the sleep features reported in this work in MCI classification, we extracted the spectral and complexity features from the awake EEG for comparison. Two classification methods were used to verify the classification effect of EEG signals during wakefulness and sleep, and the test results were evaluated and compared.

### Power Spectral Density

PSD is a frequency-dependent measure of the mean power distribution. Because EEG slowing is the main linear indicator of cognitive decline, spectral analysis is a critical parameter for measuring neurocognitive impairment (Sharma et al., 2016). Welch’s periodogram is the most generally used method for calculating the estimated value of PSD (Welch, 1967), It involves averaging sequential Fourier transforms of small windows of the signal, with or without overlapping.

The PSD was calculated every 5 min by the Welch method, using 5 s of hamming windows, with 50% overlap, and median-averaging to limit the influence of artifacts. A commonly used method of determining the window width is to adopt a window long enough to contain at least two full minimum frequency periods of interest. The lowest frequency of interest here is 0.5 Hz, so we can choose a window that is greater than or equal to 4 s. Here we chose a 5 s-long window. The following is the formula for calculating power spectral density using the Welch method:


$$\text{P}\,\left(\omega \right)=\frac{1}{\text{MUL}}\,\sum_{i=1}^{L}{\left|\sum_{n=0}^{M-1}{\text{x}}^{i}\,\left(n\right)\,{\text{d}}_{2}\,\left(n\right)\,{\text{e}}^{-j\,\omega \,n}\right|}^{2}$$

Where M is the window length, U=1M⁢∑n=0M-1d22⁢(n), $L=\frac{N-M/2}{M/2}$, the x^i^(n) is the signal for each window, and the d_2_(n) is the function of Hamming window.

### Spindle

**FIGURE 2:**
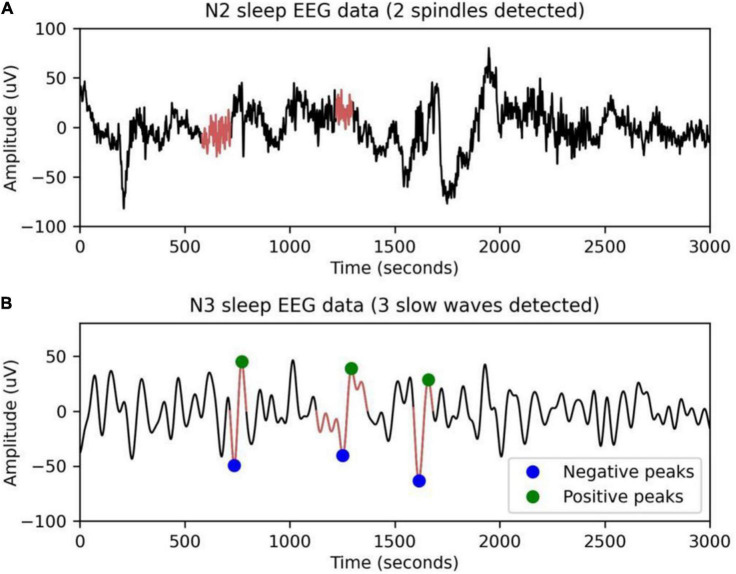
EEG waveforms detected during 30-s NREM sleep **(A)** spindles and **(B)** slow sleep waves.

Sleep spindles are a characteristic of N2 sleep, consisting of a succession of separate waves with frequencies ranging from 11 to 16 Hz (most typically 12–14 Hz), duration 0.5 s, usually using the maximum amplitude of the center deviation (Peyrache and Seibt, 2020). YASA (Yet Another Spindle Algorithm) is an open-source Python package for sleep analysis (Vallat, 2019). Spindles are detected using the YASA algorithm. The main idea of the algorithm is to calculate different thresholds from broadband filtering signals (1–30 Hz, *EEG*_*bf*_) and sigma filtering signals (11–16 Hz, *EEG*_σ_). Figure 2A shows sleep spindles as detected by a 30-s EEG segment. The algorithm consists of three steps.

Step I: The FIR filter was used to bandpass the EEG segments at 1–30 and 12–15 Hz.

Step II: Three thresholds are calculated.

Threshold 1: the relative power of the sigma band is the power of the sigma band relative to the total power of broadband (1–30 Hz). Calculated using the short-time Fourier transform (STFT), the continuous period is 2 s and the overlap is 200 ms. The first threshold is exceeded whenever the segment’s relative power is greater than or equal to 0.2.

Threshold 2: movement correlation. Pearson correlation coefficient was obtained by moving the sliding window of 300 ms and the step of 100 ms. The correlation value *r*≥0.65 will exceed the second threshold.

Threshold 3: The moving root mean square is defined by calculating the moving root mean square (RMS) of *EEG*_σ_. The window width is 300 ms and the step of 100 ms. The third threshold is exceeded whenever the RMS value of the segment *RMS*≥*RMS*_*thresh*_. Where,


$${\text{RMS}}_{\mathrm{thresh}}={\text{RMS}}_{\mathrm{mean}}+1.5*{\text{RMS}}_{\mathrm{std}}$$

Step III: Decision making. Each EEG segment detected above three thresholds is considered a potential sleep spindle. The soft threshold is calculated by smoothing the decision vector of the 100 ms window. The true start and end times of the spindles are then found in the decision vector by finding the parameters that two of the three critical values are exceeded. Finally, spindles that are close to each other (less than 500 ms) are merged, and spindles that are too short or too long are removed.

### Sleep Slow Wave

Sleep slow waves, defined as those with slow frequencies (<2 Hz) and high amplitudes (> 75 mV), have been linked to a drop in steady-state sleep pressure and the protective impact of arousal (Westerberg et al., 2012). Figure 2B shows slow sleep waves detected in 30-s EEG segments by the YASA algorithm. The algorithm consists of four steps.

Step I: Bandpass filtering is carried out between 0.3 and 2 Hz using an FIR filter with a transition band of 0.2 Hz.

Step II: Detection of all the negative peaks with amplitudes between –40 and –300 μV and all the positive peaks with amplitudes between 10 and 150 μ*V* in the filtered signal.

Step III: For each negative peak (= slow-wave trough), find the closest positive peak and calculate several metrics, including peak-to-peak (PTP) amplitude, duration of the negative and positive phases, slope, etc.

Step IV: Apply a set of logical thresholds to determine true slow waves. PTP amplitudes need to be between 75 and 400 μ*V*. The positive and negative phase duration is calculated by the zero-crossing value to ensure that the positive phase duration is 0.1–1 s and the negative phase duration is 0.3–1.5 s. The slope between the trough and the midline is greater than 0.

### Feature Extraction

We classify EEG data using four major types of features in this paper: spectral, complexity, spindles, and sleep slow waves. Table 2 shows the whole list of computed features.

**TABLE 2:** List of computed features for each 5-min segment.

Feature Group	Features
Spectral (absolute power and relative power)	Delta
	Theta
	Alpha
	Sigma
	Beta
	Gamma
	Total power
Complexity	Permutation entropy
	Singular value decomposition
	Sample entropy
	Detrended fluctuation analysis
	Petrosian FD algorithms
	Katz fractal dimension
	Higuchi’s fractal dimension
	Lempel-Ziv complexity
Spindle	Density
	Duration
	Amplitude
	RMS
	Abspower
	Relpower
	Frequency
	Oscillations
Sleep slow wave	Density
	Duration
	ValNegPeak
	ValPosPeak
	PTP
	Slope
	Frequency

For each EEG segment, absolute band power, total signal power, and relative band power are calculated using PSD. The term “relative band power” refers to the normalization of each frequency band’s power in relation to the total power of 0.5–40 Hz (Kim and Kim, 2018). A total of 13 features are spectral features. Nonlinear approaches using fractal dimension or entropy methods may facilitate the identification of MCI. We use entropy and fractal dimension methods to calculate the features of EEG segments as complexity features (Ma Y. et al., 2018). And the properties of spindles and sleep slow waves are calculated after they are successfully detected.

Spectral, complexity, spindles, and sleep slow waves features were extracted from sleep EEG. In order to test the validity of sleep features, spectral and complexity features were extracted from EEG signals during wakefulness as controls. All the features were standardized. Then, these features are then utilized to train the classifier to automatically identify between MCI and HC EEG data.

### Classification

The classifier receives the extracted features from the EEG data as input. The classifier determines which category the new observation belongs to. In this study, we use support vector machine (SVM) and gated recurrent unit (GRU) to classify the data separately.

We used two classifiers to predict the true category of subjects. The predicted outcome variable is binary (0 for healthy controls, 1 for patients with MCI), and the predicted scores range from 0 to 1. The predicted scores of the subjects were utilized as MCI scores. All data sets were randomly separated into two divisions, 80% of the data were used for training and 20% for testing. The MCI classification model was fitted using the training data. We fitted the MCI classification model on the whole training data using the optimal hyperparameter configuration to determine the performance of the MCI classification model in the training data. These optimal hyperparameters were determined using 10-fold cross-validation. The generated model was directly applied to the test data, and the MCI score was used to determine whether the subject was HC or MCI. We use two classifiers to perform MCI detection respectively, in order to analyze detection results according to the structure of different types of classifiers.

#### Support Vector Machine

The SVM classifier is a supervised learning approach for separating two classes by finding the best separation hyperplane in the feature space (Safi and Safi, 2021). For N training samples {(*x*_*i*_,*y*_*i*_),*i* = 1,⋯,*N*}, where *x_i_* is the *i* th input vector and *y_i_* is the known target, SVM training is the same as figuring out how to solve the following optimization problem:


$$\min_{w,b,\xi }\text{J}\,\left(w,\xi \right)=\frac{1}{2}\,{\text{w}}^{T}\,\text{w}+\text{c}\,\sum_{i=1}^{N}\xi_{i}$$

subject to:


$${\text{y}}_{i}\,\left[{\text{w}}^{T}\,\varphi \,\left({\text{x}}_{i}\right)+\text{b}\right]\geq 1-\xi_{i},\xi_{i}\geq 0$$

Where ξ_*i*_ is slack variable, indicating the tolerance of misclassification. C is a punishment parameter that is used to penalize mistakes during training, *b* is a bias term, *w* is the weight applied for input data *x_i_*. The kernel function φ(*x*) is a nonlinear transformation function that maps the input vectors into a high-dimensional feature space (Madusanka et al., 2019).

#### Gate Recurrent Unit

GRU is a variation structure of the Recurrent Neural Network (RNN). RNN will remember past information and apply it to the current output computation. Furthermore, RNN suffers from the problem of vanishing and exploding gradients (Chung et al., 2014), which causes the model to learn and train slowly. These concerns are addressed by taking into account its versions, such as GRU, which works on gated mechanisms. A GRU has two gates, the update gate controls how much prior state information is brought into the present state, while the reset gate controls how much previous state information is ignored. The following expressions show how a GRU calculates the result.


$$r=\sigma \left(wv_{r}i_{t}+xrhs_{t-1}\right)$$

$$u=\sigma \left(wvui_{t}+xrhs_{t-1}\right)$$

$$h_{t}=tanh\left(wvi_{t}+x\left(r⊙hs_{t-1},x_{t}\right)\right)$$

$$h_{st}=\left[\left(1-u\right)hs_{t-1}\right]+uh_{t}$$

where, *r* is a reset gate and *u* is the update gate, σ is the sigmoid function, *h_t_* for the hidden state, element multiplication denoted by ⊙.

Bidirectional GRU (Bi-GRU) can not only take advantage of past information, but also capture subsequent information (Ma C. et al., 2018).


$${\text{h}}_{t}=\left(\vec{{\text{h}}_{t}}||\overset{\leftarrow }{{\text{h}}_{t}}\right)$$

where, *h_t_* for output states, $\overset{\leftarrow }{h_{t}}$ a backward and $\vec{h_{t}}$ forward states in the opposite direction.

### Performance Evaluation

To decide which classifier method is the most successful, the sensitivity, specificity, F1 score, and accuracy of each one should be calculated. The confusion matrix gives an exact idea of the number of correctly classified and unclassified samples. The parameters are calculated by the following equations:


$$Sensitivity=\frac{TP}{TP+FN}*100\%$$

$$Specificity=\frac{TN}{TP+FN}*100\%$$

$$Accuracy=\frac{TP+TN}{TP+FN+TN+FP}*100\%$$

$$F_{1}=2*\frac{precision*recall}{precision+recall}$$

## Results and Discussion

According to the steps of this method, EEG segments of 40 different subjects with HC and MCI were analyzed. Then, in the analysis step, the features of the signals during wakefulness and NREM sleep are extracted, respectively. For the training of the SVM classifier and the GRU network, all datasets were randomly separated into two divisions of the training set and testing set for evaluating the accuracy of the classifiers, with 80 percent of the data treated as training data and 20% of the data considered as testing data. The test accuracy, sensitivity, specificity, and F1 score were finally acquired with 10-fold cross-validation was used to find the best hyperparameters values.

### Results

To construct SVM classifiers, the “fitcsvm” module of MATLAB is used. Bayesian hyperparameter optimization is used to find the hyperparameter that minimizes the cross-validation loss to optimize the classifier. In this study, the cubic polynomial kernel function was selected, gamma = 2.15/C = 1 for the features of NREM sleep and gamma = 13.2/C = 2 for the features of wakefulness was used to obtain the best results. GRU classifier construction, training, and testing were carried out with the assistance of the PyTorch library. The default GRU sequential class was updated to add different layers based on the models provided. For two GRU networks, we used batch size 32, Adam optimizer, and binary cross-entropy loss, and varied dropout, and the number of hidden layers using random search (Yasir et al., 2021). For the features of NREM sleep, the best results for GRU were achieved with the dropout parameter value 0.5, the output layer has 2 nodes, the input layer has 36 nodes, and finally three hidden layers have 89 nodes. For the features of wakefulness, the best results for GRU were achieved with the dropout parameter value 0.5, the output layer has 2 nodes, the input layer has 21 nodes, and finally three hidden layers have 59 nodes. We trained 200 epochs for GRU and tested on the epoch that had the best cross-validation accuracy.

The mean of each metric was used to objectively evaluate classification performance, and the classification results comparison between EEG during NREM sleep and wakefulness are shown in Table 3.

**TABLE 3:** Classification performance comparison between EEG during NREM sleep and wakefulness for distinguishing MCI from HC.

	Classifier	Accuracy (%)	Sensitivity (%)	Specificity (%)	F1 score (%)
W	Cubic SVM	85.47	88.57	82.14	84.35
	Bi-GRU	90.26	90.00	90.54	90.57
NREM	Cubic SVM	90.51	92.02	88.89	89.87
	Bi-GRU	93.46	93.33	93.60	93.56

As shown in Table 3, for different feature sets, the classification accuracy of the sleep EEG feature set is higher than that of the awake EEG feature set, and the sensitivity, specificity, and F1 score are also higher than that of the awake EEG. The results of the two classifiers showed that sleep EEG had better performance in MCI and HC classification than awake EEG, and the common features of sleep and awake EEG combined with sleep features could significantly improve the classification accuracy. For different classifiers, the accuracy of the GRU network is 5 and 3% higher than that of the SVM classifier for the same features, indicating that the classification performance of GRU is better than that of the SVM classifier. These results suggest that sleep features can reflect cognitive performance in patients with MCI and emphasize that altered sleep is a component of mild cognitive impairment.

**FIGURE 3:**
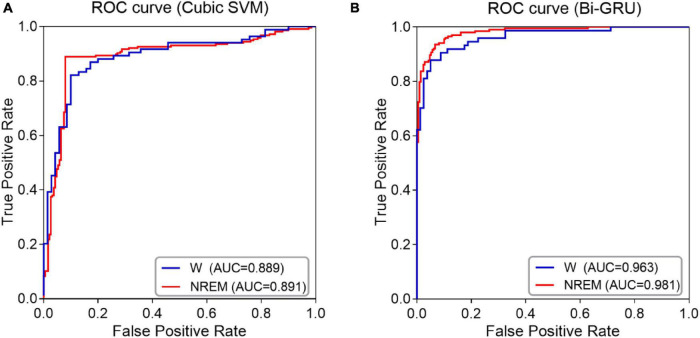
ROC curves and AUC values of NREM sleep EEG and awake EEG **(A)** SVM classifier **(B)** GRU network.

The classification performance was also confirmed by ROC curve analysis, especially by calculating the area under the ROC curve (AUC). As shown in Figure 3, when awake and sleep EEG features are used as inputs to the classifiers, ROC curves and corresponding AUC values for MCI and HC classification are calculated. The ROC curve of sleep EEG was closer to the upper left corner, and the AUC value of GRU was higher than that of awake EEG, indicating that the GRU network with sleep EEG feature input achieved the best performance (AUC value was as high as 0.981).

The results showed that the GRU classifier with the sleep features had the best effect, with an accuracy of 93.46%, sensitivity of 93.33%, specificity of 93.60%, F1 value of 93.56%, and AUC value of 0.98. In conclusion, the spectral and complexity analysis of sleep EEG, combined with the features of sleep slow waves and spindles, and the classification of sleep EEG by GRU are effective for the early detection of MCI.

### Discussion

AD is an irreversible neurodegenerative disease, so early screening and diagnosis of MCI is particularly important, which is the key to effective early intervention of AD and delay the progression of dementia. MCI is currently diagnosed by specialists through extensive testing, including neurophysiological assessment, blood analysis, cerebrospinal fluid analysis, and imaging techniques. However, the evaluation of these medical records is not only costly and complex to implement, but also requires experienced physicians. Therefore, the automated decision method which only needs one physiological parameter can not only objectively evaluate the patients, but also ensure high diagnostic accuracy. In addition, it will be economical, portable, and more suitable for the elderly population.

Several innovative research in recent years have only focused on awake EEG to detect MCI, and they collected about 20 channels of EEG and extracted features for classification. The DWT decomposition method was utilized to analyze 20 channels of EEG signals, and the Hjorth parameter and KNN classifier were incorporated to achieve 97.64% accuracy (Safi and Safi, 2021). Although this method has high classification accuracy, the acquisition channels of the EEG signal are numerous, and the measurement method is complex. Khatun et al. (2019) proposed an MCI detection method based on single-channel EEG, which stimulated auditory speech signals, extracted features from event-related potential (ERP), and obtained an accuracy of 87.9% by SVM classification. This method uses a single-channel signal, but it requires the collection of evoked potentials, which is complicated and difficult for patients to cooperate with. Cejnek et al. (2021) proposed a new approach for detecting MCI based on EEG recordings. The highest accuracy was 91.62 percent among the results of cross-validation classification between HC and MCI patients by each channel. All of the work above on EEG acquisition methods relied on the analysis of EEG recordings of waking states. It is worth noting that in this study, we proposed using the features extracted from sleep EEG to detect MCI and achieved an accuracy of 93.46%. Studies have shown that changes in sleep electrophysiology may be linked to the cognitive condition of AD and MCI patients. We used spindles and sleep slow waves, along with other common features, to improve classification accuracy by the GRU network. Although the accuracy is lower than that of multi-channel EEG signal classification, it is superior to other single-channel results and is not limited by the experimental site. Therefore, it is especially suitable for head-mounted wearable devices.

**FIGURE 4:**
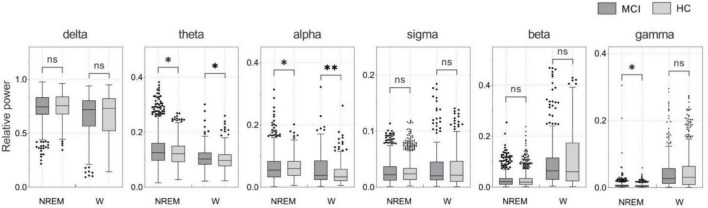
Relative power of NREM sleep and awake EEG in MCI and HC subjects (*p < 0.05; ^**^p < 0.01).

In this study, we proposed a diagnostic method for patients with MCI based on sleep EEG signals. We have shown that incorporating features of sleep slow waves and spindles as new features of the MCI detection significantly improves the accuracy of the MCI detection over traditional features during wakefulness. The proposed method is 5 and 3% better than traditional features in the classification results of the SVM classifiers and GRU network, respectively. In this study, the detection of MCI using the GRU network with the features of NREM sleep achieved the highest accuracy, reaching 93.46%. The following three aspects are considered superior to other methods. Firstly, the features of spindles and slow sleep waves during NREM sleep are an effective supplement to traditional features, and the data during sleep contains more information than wakefulness. As shown in Figure 4, SPSS software was used for statistical analysis. Non-normally distributed variables were compared by the Mann-Whitney U test. *P*-values less than 0.05 were considered statistically significant. During NREM sleep, the MCI group showed significant differences in theta, alpha, and gamma bands compared with the HC group. During wakefulness, a similar phenomenon was observed, but only the theta and alpha bands were significantly different (both *p* < 0.05). This suggests that significant changes in power spectral density can be detected during both NREM sleep and awake sleep, and that sleep EEG does not reduce the difference between MCI and HC. Therefore, related features of sleep may be an important biomarker of MCI. Secondly, this algorithm adopted two-channel EEG, which is simple compared with the multi-channel acquisition, and does not require additional nursing staff to take care of patients when we collect the EEG signals during sleep. Finally, the GRU network significantly improved the classification performance in MCI recognition.

While the proposed approach is encouraging, there are some limitations that should be addressed. In this study, only MCI and HC were categorized, requiring further consideration of other stages of cognitive impairment. In addition, we were only able to analyze sleep EEG signals from 40 different subjects in this study, so this research should be regarded as preliminary, and future studies should include larger datasets to validate the suggested method’s stability and generalizability.

## Conclusion

EEG signals are non-stationary, nonlinear, and noisy, so it is a challenging problem to distinguish between MCI and HC based on EEG signals. In this study, MCI was detected and classified using two-channel sleep EEG signals. Based on traditional features, the features of sleep slow waves and spindles were extracted. Unlike the existing features, the proposed features are not restricted by the use of traditional EEG bands. In particular, sleep features combined with traditional features outperformed traditional features on classification tasks, proved to be more accurate in predicting MCI and performed better with sleep EEG signals than with wakeful signals. Although studies have shown that changes in EEG activity during NREM sleep are associated with MCI, no studies have used it for the recognition of MCI. The high classification accuracy obtained in this paper once again proves that sleep slow waves and spindles can be used as early biomarkers for the development of AD (Romanella et al., 2020). Early diagnosis would also provide patients access to available treatment, while possibly initiating an earlier treatment. In addition, this study is based on sleep EEG, which is non-invasive, portable, and low-cost, and therefore has high value as a diagnostic tool.

## Data Availability Statement

Publicly available datasets were analyzed in this study. This data can be found here: https://sleepdata.org/datasets/sof; Study of Osteoporotic Fractures.

## Ethics Statement

The study was conducted using an open source database. The procedures for information collection in the database were in accordance with the ethical standards of the institutional and national Non-invasive Clinical Research Medical Ethics Review Board. Ethical review and approval was not required for the study on human participants in accordance with the local legislation and institutional requirements. Written informed consent for participation was not required for this study in accordance with the national legislation and the institutional requirements.

## Author Contributions

DG and CW were responsible for the study concept and design. DG and ZF contributed to the acquisition of data and funding. CW determined software and analytical methods as well as manuscript writing. YZ assisted with data analysis and interpretation of findings. KY and HA provided critical revision of the manuscript for important intellectual content. All authors critically reviewed the content and approved the final version for publication.

## Conflict of Interest

The authors declare that the research was conducted in the absence of any commercial or financial relationships that could be construed as a potential conflict of interest.

## Publisher’s Note

All claims expressed in this article are solely those of the authors and do not necessarily represent those of their affiliated organizations, or those of the publisher, the editors and the reviewers. Any product that may be evaluated in this article, or claim that may be made by its manufacturer, is not guaranteed or endorsed by the publisher.
